# Mental Health and Malocclusion: A Comprehensive Review

**DOI:** 10.3390/clinpract15030044

**Published:** 2025-02-24

**Authors:** Osama A. Alsulaiman, Maha I. Alghannam, Dalal M. Almazroua, Abdulaziz S. Alamri, Suliman Y. Shahin, Essam A. Nassar, Naif N. Almasoud, Abdulrahman T. Alsulaiman, Ahmed A. Alsulaiman

**Affiliations:** 1 Department of Preventive Dental Sciences, College of Dentistry, Imam Abdulrahman Bin Faisal University, Dammam 32222, Saudi Arabia; oaalsulaiman@iau.edu.sa (O.A.A.); dmalmazrou@iau.edu.sa (D.M.A.); absalamri@iau.edu.sa (A.S.A.); sshahin@iau.edu.sa (S.Y.S.); enassar@iau.edu.sa (E.A.N.); nnalmasoud@iau.edu.sa (N.N.A.); 2 Department of Dental Education, College of Dentistry, Imam Abdulrahman Bin Faisal University, Dammam 32222, Saudi Arabia; mialghannam@iau.edu.sa; 3 Department of Maxillofacial and Oral Health, King Fahad Hospital of The University, Imam Abdulrahman Bin Faisal University, Dammam 32222, Saudi Arabia; atsulaiman@iau.edu.sa

**Keywords:** skeletal malocclusion, dental malocclusion, psychological impact, mental health, depression, anxiety

## Abstract

The purpose of this study is to comprehensively review the relationship between malocclusion and anxiety and depression. While the physical implications of malocclusion are well documented, recent scholarship has shifted focus to examining the direct relationship between malocclusion and both anxiety and depression. It has been hypothesized that individuals with skeletal or dental malocclusion experience a range of psychological sequelae, including diminished quality of life (QoL), reduced oral-health-related quality of life (OHRQoL), increased vulnerability and appearance-related bullying, and impaired body image. Furthermore, these factors are postulated to collectively contribute to overall mental health, with malocclusion potentially serving as a contributing etiological factor in the development of elevated levels of anxiety and depression. Contemporary scholarship has established a complex relationship between dentofacial deviations and the psychological well-being of affected individuals. Evidence shows that malocclusion may contribute to increased depression and anxiety levels in some individuals, influencing their social functioning and treatment-seeking behavior. Dentofacial disharmony has also been associated with altered self-perception, potentially impacting an individual’s OHQOL and overall quality of life. While the findings exhibit some inconsistency, a modest body of evidence indicates a possible correlation between pronounced skeletal or dental malocclusion and anxiety and depression. These adverse psychosocial impacts, in turn, contribute to an elevated risk of anxiety and depression, underscoring the far-reaching consequences of malocclusion beyond oral health. Therefore, clinicians need to consider these issues in their treatment plans, incorporating interdisciplinary approaches that address both orthodontic and psychological aspects of patient care.

## 1. Introduction

Malocclusion has wide health implications. The World Health Organization (WHO) classifies this highly prevalent public health problem [1,2] as a “handicapping dentofacial anomaly” that has adverse effects on orofacial functionality, dentofacial morphology, and psychosocial well-being [3,4,5]. While the physical implications of malocclusion, such as difficulties in mastication, speech impediments, and increased risk of dental trauma, are well documented [6], its psychological ramifications have gained increasing attention in recent years. The impact of malocclusion extends beyond oral health and can profoundly influence an individual’s psychological well-being. In fact, there is an inverse correlation between the severity of skeletal or dental malocclusion and psychological implications across different age groups [7,8,9].

The oral-facial region often becomes a major focus for individuals because it attracts the most attention during interpersonal interactions and serves as the main channel for physical, emotional, and vocal communication [10]. Therefore, facial aesthetics significantly impact one’s social interactions and interpersonal evaluations [11], with dental features significantly contributing to overall facial appearance and body image perception [12]. Consequently, visible dentofacial deformities can lead to a cascade of psychological effects from mere aesthetic concerns to issues regarding body perception [5], self-esteem [13,14], health-related quality of life, particularly in terms of psychological discomfort and disability [15,16], and social stigma, including peer victimization [17]. Empirically, individuals with malocclusion exhibit traits such as low self-esteem and psychological distress [17], factors that significantly influence social perceptions, personal experiences, and psychological well-being [18]. Therefore, the impact of malocclusion underscores a critical need for comprehensive orthodontic interventions that address not only the physical and functional aspects of malocclusion but also its profound psychosocial ramifications [19,20,21].

Malocclusion, a term denoting significant deviations from optimal occlusal relationships, encompasses misalignments of dental structures, maxillofacial skeletal components, and associated soft tissues [22,23]. Moreover, malocclusion may be classified as either skeletal (caused by jaw discrepancies, e.g., Class II/III malocclusions with convex/concave profiles) or dental (resulting from tooth misalignment, e.g., crowding, spacing, or Class I malocclusion). While skeletal malocclusion involves the underlying bone structure, dental malocclusion pertains to tooth positioning, though overlapping presentations may occur [24]. Several environmental factors, oral habits, social traits, and genetic predispositions have been associated with its development [25,26]. Also, malocclusion has been linked to gender differences, with varying prevalence rates/impacts among different demographic groups, further emphasizing the complex interplay between malocclusion and psychological factors [27,28]. For instance, class II skeletal malocclusions are more common than class III malocclusions, with each type presenting its own unique challenges both dentally and skeletally [29]. Aesthetic improvements resulting from orthodontic treatment can lead to enhancements in oral-health-related quality of life, as well as reduced psychological discomfort and disability [30]. Empirical evidence strongly supports the positive impact of orthodontic treatment on patients’ psychological well-being and self-esteem. A systematic review [31] revealed that 37.5% of studies reported significant increases in self-esteem after orthodontic treatment. Similarly, corroborating these results, a separate clinical study focusing on adult patients undergoing oral rehabilitation observed a statistically significant elevation in self-esteem scores post-orthodontic treatment, as measured by the validated Rosenberg self-esteem scale [32]. The same study reported marked improvements in OHRQoL, assessed using the Oral Health Impact Profile (OHIP-14). In addition to this, orthognathic surgery can positively impact patients’ psychology and self-esteem, particularly in cases of skeletal Class III malocclusion [33]. Both anxiety and depression have been associated with stress-related mental health disorders [34].

Given that skeletal/dental malocclusion can decrease self-esteem and promote psychological distress, we aim to study the association between dentofacial conditions and psychological implications with a particular focus on anxiety and depression. We hypothesized that patients with skeletal/dental malocclusion are prone to anxiety and depression with varying degrees. With that, our two-fold objective includes, firstly, to comprehensively review the existing literature on the relationship between skeletal or dental malocclusion and mental health in the form of depression and anxiety; and secondly, to identify gaps in current research and propose directions for future studies in this field. Therefore, the aim of this study is to comprehensively review the relationship between malocclusion and anxiety and depression.

## 2. Materials and Methods

### 2.1. Study Design

We conducted a comprehensive literature review using Preferred Reporting Items for Systematic Reviews and Meta-Analyses (PRISMA) guidelines [35] for the procedural framework. Adding to that, a narrative review with a critical evaluation of the literature was performed following the Joanna Briggs Institute (JBI) methodology while utilizing their checklists [36,37]. We styled our research after a narrative review approach [38]. Narrative approach is particularly helpful in broader explorations of complex relationships [39], and this is exactly what may be needed in exploring correlation between anxiety or depression and skeleto-dental malocclusion. This style of research also facilitates a subjective examination of the literature, providing insights that are not constrained by rigid methodological protocols [40]. This is particularly valuable in this under-researched area. And lastly, narrative approach is effective when it comes to identifying the gaps in the existing literature, thereby prompting future research questions [41].

### 2.2. Eligibility Criteria

To be included in narrative review, randomized controlled trials (RCTs), non-randomized intervention studies, observational studies (including cohort, case-control, and cross-sectional studies), and qualitative research needed to fulfil the following criteria: (1) examine the relationship between malocclusion and psychological outcomes and oral health at a single point in time; (2) use validated instruments to measure malocclusion, like the Dental Aesthetic Index (DAI) or the Index of Orthodontic Treatment Need (IOTN); and (3) have experimental/control groups. In addition, investigations needed to use qualitative research methodologies/standardized psychometric tools to assess psychological impacts. Moreover, studies published in English only were eligible to be included.

The following exclusion criteria were applied to maintain focus on the psychological impact of malocclusion: (1) studies solely examining physical or functional outcomes without psychological assessment were omitted; (2) investigations centered on orthodontic treatment techniques without consideration of patient psychology were excluded; (3) research primarily assessing clinical outcomes without exploring psychological implications was disregarded; (4) and studies focusing on surgical interventions for malocclusion without paying specific attention to psychological factors were excluded. Additionally, case reports and non-peer-reviewed literature were omitted to ensure scientific rigor.

### 2.3. Literature Search

The process of systematically searching for acceptable publications was conducted on 27 March 2024. The searches were completed for articles published between 1 January 1990 and 30 September 2024 and covered PubMed, PubPsych, Web of Science (WoS), and Cochrane reviews. Additionally, grey literature was scrutinized to capture relevant non-indexed publications. To obtain the best search results, the following search string was constructed and employed across the aforementioned databases: (malocclusion OR “dental occlusion, traumatic” OR “tooth abnormalities” OR “dental occlusion” OR “bite, cross” OR “bite, open” OR “overjet” OR “overbite”) AND (“psychological impact” OR “mental health” OR depression OR anxiety “emotional well-being” OR “psychological distress” OR “psychosocial factors” OR “psychological adaptation” OR stress OR “mental disorders”). The psychological component of the research encompassed both general terms like “mental health” and specific conditions such as depression and anxiety, as well as broader concepts like quality of life and self-esteem.

### 2.4. Data Extraction

The following details were obtained via a standard data form: first author’s name, year of study, country of origin, study design, employed methods, characteristics of the patients (age, anxiety or depression measurements, and results before and after treatment), and conclusions.

### 2.5. Joanna Briggs Institute (JBI) Criteria for Quality Analysis

To assess the methodological quality, each study was critically evaluated using the checklists designed by the JBI [36,37]. Cohort studies were evaluated using the 11-item JBI tool specifically designed for cohort studies [36]; cross-sectional studies were evaluated via 8-item JBI critical appraisal checklist for analytical cross-sectional studies [42]; and pre-test–post-test studies were evaluated using the 9-item JBI tool for quasi-experimental studies [36]. For every study, each criterion was assessed as either “Yes” (indicating that the criterion was met), “No” (indicating that the criterion was not met), “Unclear” (when there was insufficient information), or “Not applicable” (when the criterion was not relevant). The risk of bias was then classified based on the proportion of “Yes” responses to the total number of applicable criteria. For quasi-experimental studies, meeting 78–100% of the criteria indicated a low risk of bias, while 44–77% suggested a moderate risk, and 0–43% indicated a high risk. Similarly, for cross-sectional studies, 75–100% denoted a low risk, 38–74% represented a moderate risk, and 0–37% signified a high risk. For cohort studies, 73–100% corresponded to a low risk, 36–72% indicated a moderate risk, and 0–35% suggested a high risk. It is important to note that “Unclear” responses were conservatively considered as not meeting the criteria, while “Not applicable” responses were excluded from our calculations.

## 3. Results

### 3.1. Study Selection

The search yielded a total number of 1842 studies from electronic databases, which were exported to Rayyan^®^, a specialized systematic review management software [43], for the first stage of screening. Among these records, 279 were Cochrane reviews, 413 came from PubMed, 198 were retrieved in PubPsych, 305 appeared in Scopus, 322 came from Web of Science, and 154 items were found on Google Scholar. A separate 171 records were shortlisted through grey literature and a manual search of reference lists. Following the removal of duplicates and an initial review of the titles and abstracts, 893 publications were screened based on their titles and abstracts. Among these publications, 111 underwent manual screening and a discussion with a third reviewer, resulting in the exclusion of 782 records. A total of 70 studies were excluded because they were either not what we sought, or did not meet our methodological constraints, or had irrelevant outcomes.

In the end, forty-one were shortlisted, including twenty-five anxiety-related studies (four of which are animal models and ten of which are human clinical trials), eleven depression-related studies (one of which is an animal study and two are human randomized controlled trials), and five studies with both anxiety and depression as themes. Finally, 11 studies were eligible for inclusion and quality assessment and consequently were included in the review. These narrow search criteria are justified by the specificity of the research objective, which exclusively targeted anxiety and depression linked to skeletal or dental malocclusion, deliberately excluding broader psychological domains. Furthermore, the stringent exclusion criteria systematically filtered out studies lacking psychological assessment or those focusing solely on clinical, surgical, or functional outcomes, ensuring strict thematic alignment. Cross-database searches and rigorous screening minimized selection bias while maintaining methodological rigor, reflecting the niche focus of the review.

**Figure 1:**
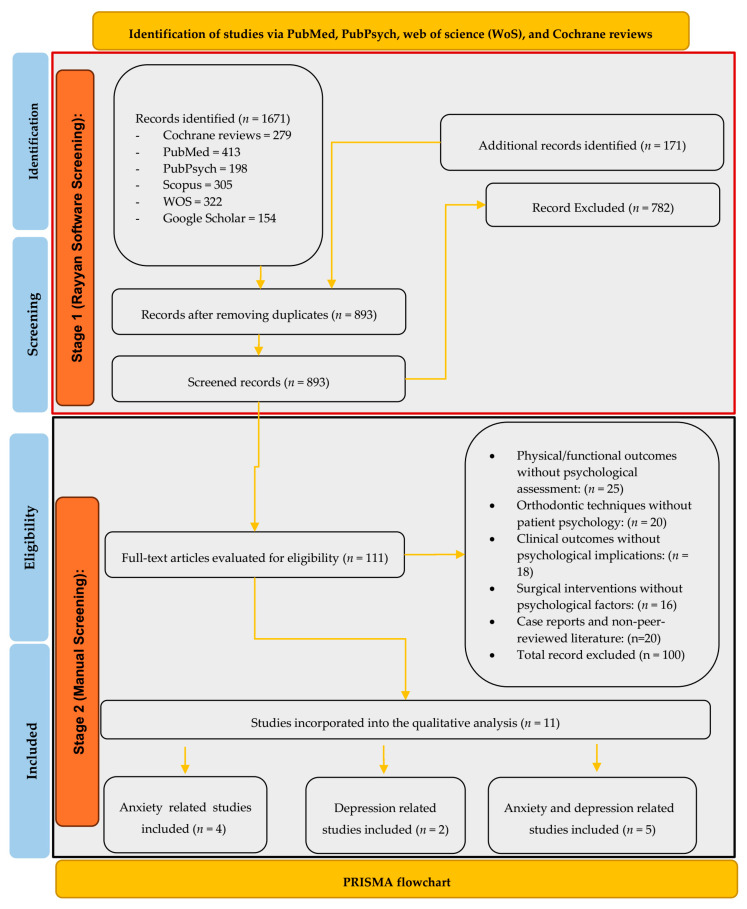
PRISMA flow diagram of study selection.

Two independent reviewers (AA and OS) critically assessed the quality of studies. In the case of a disagreement, this was settled through a discussion or, if necessary, a third reviewer (NM) was engaged. Cohen’s Kappa statistics were computed to assess the inter-rater reliability [44]. The degree of agreement between the two raters (AA and OS) with respect to the title and abstract was found to be strong, as evidenced by the inter-rater reliability, which demonstrated a concordance rate of 99.30% and Cohen’s Kappa (k) value of 0.79. In Figure 1, there is a PRISMA flowchart illustrating the research process, which is divided into two main stages—stage one corresponds to screening via Rayyan software (a web application with no public version) while stage two corresponds to the manual screening of the studies.

### 3.2. Quality Analysis

**Figure 2:**
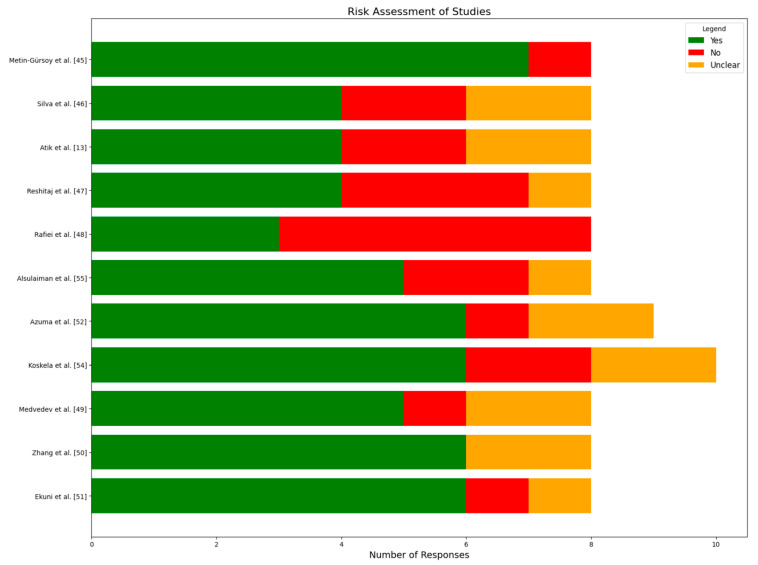
Detailed risk assessment of included studies [13,45,46,47,48,49,50,51,52,54,55].

Regarding the study design, the included articles were eight cross-sectional studies [13,45,46,47,48,49,50,51], two were quasi-experimental studies [52,53], and one cohort study [54]. Table 1, Table 2 and Table 3 provide quality appraisals according to the JBI criteria. The studies assessed reveal a distribution of risk levels, with 10% (1 out of 11) categorized as low risk by Metin-Gürsoy et al. [45]. Significantly, 81.81% (9 out of 11) of the studies, including works by Silva et al. [46], Atik et al. [13], Reshitaj et al. [47], Medvedev et al. [49], Zhang et al. [50], Ekuni et al. [51], Azuma et al. [52], Koskela et al. [54], and Alsulaiman et al. [55], fall into the moderate-risk category, indicating a moderate level of reliability. Only 10% (1 out of 11) of the studies, represented by Rafiei et al. [48], are classified as high risk, suggesting lower reliability. This distribution underscores that the majority of the studies demonstrate a moderate risk, with fewer instances of low- and high-risk studies. Figure 2 displays a detailed risk assessment of the studies.

### 3.3. Anxiety and Malocclusion

Anxiety is an emotional state and frequently occurs before encountering a perceived threat, even if the threat is unclear [56]. It is a common occurrence in everyday life and is usually accompanied by symptoms such as restlessness, weariness, difficulty concentrating, irritability, muscle tension, and sleep difficulties [56,57]. To be diagnosed with generalized anxiety disorder (GAD), these symptoms must last at least six months and produce significant disruption in social, occupational, or other key elements of daily life [58]. Anxiety can lead to responses that are cognitive, emotional, physical, and behavioral. Several commonly used tools are available for measuring anxiety, each with a distinct emphasis. The Spielberger state-trait anxiety inventory (STAI) [59] distinguishes between transient state anxiety and enduring trait anxiety. The Beck anxiety inventory (BAI) [60] evaluates the severity of anxiety symptoms, while the Hamilton anxiety rating scale (HAM-A) [61] assesses both the psychological and physical aspects of anxiety. The Generalized Anxiety Disorder 7 (GAD-7) assessment [62] is a brief screening tool for generalized anxiety disorder, and the hospital anxiety and depression scale (HADS) [63] measures anxiety and depression, particularly in clinical settings. While these tools have demonstrated reliability and validity across multiple languages, no instrument is universally accepted as the gold standard due to inherent limitations [56]. As of 2019, around 13% of the global population experiences mental health issues [64], with anxiety and depressive disorders being the most common causes of disease and disability.

Studies reveal that many people experience anxiety about their dental appearance at some point in their lives, leading to feelings of worry, fear, nervousness, or apprehension [56,65]. Such individuals may also develop strategies like hiding their teeth and avoiding smiling, which can result in social anxiety, emotional insecurity, fear, and difficulties in personal relationships [66]. Moreover, anxiety levels about self-image and self-esteem could vary from mild to severe [67]. The occurrence of major anxiety disorders notably becomes more frequent from late adolescence or early adulthood [68,69], as during this stage individuals undergo significant and rapid transformations in their cognitive, social, and physical development [70].

#### Correlation Between Malocclusion and Anxiety

Both in animal as well as in human model settings, it has been observed that malocclusion positively correlates with anxiety. In an animal model setting, a significant correlation between malocclusion and anxiety disorders has been observed [71]. The placement of acrylic caps on the lower incisors of rats and bite raising in aged mice has been found to trigger increased plasma corticosterone levels [72,73]. A neurological pathway linking dental misalignment to both physical discomfort and anxiety has been found [74]. Similarly, it has been concluded that malocclusion, specifically unilateral anterior crossbite (UAC), can lead to TMD and anxiety-like behaviors in rats. Another study [75] contributes to the understanding of the neurobiological basis of malocclusion-induced anxiety. The authors’ findings indicate that UAC induces anxiety-like behaviors in mice.

This relationship is mediated through a neural pathway involving the ventral posterior medial nucleus of the thalamus (VPM) and vesicular glutamate transporter 1 (VGLUT1) neurons in the ventral posterior division of the medial part of the spinal trigeminal nucleus (Vpdm).

Conversely, a modest body of literature suggests a potential association between skeletal/dental malocclusion and the prevalence of anxiety in humans. Table 4 compares four cross-sectional/observational studies that were carried out in Brazil, Turkey, and Kosovo between 2019 and 2023, investigating, among various other psychological issues, the relationship between malocclusion and anxiety in diverse populations ranging from schoolchildren to young adults. Of the four studies reviewed, three focused on dental malocclusion (e.g., crowding/spacing and caries-related misalignment (Refs. [45,46,47])), while one [13] addressed skeletal malocclusion. Although the results show that there might be a link between malocclusion and psychological distress, there is a scarcity of data about the impact of mild to moderate malocclusions on anxiety. No clear correlation emerged from the studies reviewed. Some of the studies [13,45] reported a significant positive correlation between malocclusion severity and anxiety levels. To this end, Atik et al. [13] found out that individuals with more severe malocclusions exhibited higher anxiety scores, suggesting that dental appearance significantly affects general anxiety. However, the authors of other studies [47,52] found contrasting results. Reshitaj et al. [47], for instance, found lower dental anxiety in children with malocclusion compared to those with dental caries, indicating that malocclusion may cause less anxiety than other dental conditions. Azuma et al. [52] reported no significant differences in anxiety levels among groups with varying malocclusion severity levels. This disagreement may be attributed to the multifaceted nature of anxiety, which can be influenced by various factors beyond dental appearance. Moreover, the inconsistency in findings could be due to differences in study populations, assessment methods, and cultural contexts. Some studies such as [46] highlighted the potential impact of age and gender on the relationship between malocclusion and anxiety, with girls and older adolescents potentially experiencing stronger effects. So, there needs to be more targeted research in this area. Nevertheless, significant evidence also suggests that dentofacial treatment leads to reduced symptoms of anxiety [52,76,77].

### 3.4. Depression and Malocclusion

Depression is a severe psychological disorder that encompasses persistent feelings of sadness, hopelessness, and a lack of interest in, or enjoyment of, activities [81,82,83]. This condition has a profound impact on cognitive, emotional, and physical well-being, leading to significant impairment in daily functioning across social, occupational, and personal domains. The World Health Organization (WHO) estimates that approximately 5% of adults worldwide suffer from depression, with a higher incidence among women and older individuals. Studies like [53] show that chronic stress can cause neuronal damage and depression. Self-report and clinician-administered measures are important methods for depression evaluation in clinical practice as well as research. Self-report measures, as they have existed in cognitive psychology and clinical science for a long time and were initially derived from subjective evaluations of affect, cognition, and behavior—are particularly attractive given their efficiency compared to more psychometrically mature instruments like diagnostic interviews. For example, both the Beck Depression Inventory (BDI) [84] and Patient Health Questionnaire-9 (PHQ-9) [85] give quantifiable metrics of depression severity and enable longitudinal tracking. Clinician-administered assessments, such as the Hamilton Depression Rating Scale (HAM-D) [86] and the Structured Clinical Interview for DSM-IV Axis I Disorders (SCID-I) [87], provide comprehensive evaluations via structured interviews or observer-rated scales, enhancing diagnostic precision. In addition to DSM-defined measures, population-specific scales for older adults, such as the Geriatric Depression Scale (GDS) [88] and the Children’s Depression Inventory (CDI), have been developed.

#### Correlation Between Malocclusion and Depression

There is a deficiency of research examining the direct relationship between malocclusion (i.e., as a predictor variable) and depression (i.e., as an outcome variable). To the best of our knowledge, in a human model setting, there are only two studies [48,55] that directly attempt to establish a correlation between depression and malocclusion. Table 5 presents both studies. Rafiei et al. [48] reported a notable prevalence of depression (28.3%) among orthodontic patients aged 16–29 years. Class III malocclusion patients exhibited the highest prevalence (39.6%) compared to Class I (22.4%) and Class II (32.7%) (*p* = 0.003). The absence of significant associations between depression and demographic factors such as gender, marital status, or educational status (*p* > 0.05) further emphasizes the potential role of malocclusion in depressive symptomatology. These findings collectively suggest that malocclusion, particularly severe forms like Class III, may have broader implications for mental health than previously recognized. On the other hand, research by Alsulaiman et al. [55] showed that logistic regression models found no significant association between malocclusion traits and major depressive episodes (MDEs). However, the models revealed a positive association between malocclusion traits (specifically, upper crowding and two or more cumulative malocclusion traits) and dysthymia. It is imperative to note that while these studies establish a correlation, causal relationships cannot be definitively inferred without further longitudinal and interventional research.

### 3.5. Anxiety, Depression, and Malocclusion

There is a modest body of research that has attempted to find the correlation between both anxiety and depression and malocclusion as well as other psychological factors, such as QoL, self-esteem, self-image, and so on. Koskela et al. [54] found no statistically significant difference in anxiety and depression rates between adolescents with severe malocclusion and controls using the TPI and standardized psychological assessments. Conversely, Medvedev et al. [49] reported elevated rates of anxiety and depression symptoms in both cleft and non-cleft patients with skeletal malocclusions compared to controls, utilizing the IOTN-DHC and HADS. Zhang et al. [50] observed significantly higher anxiety and depression scores in patients with Class I, II, and III malocclusions compared to those with normal occlusions (*p* < 0.001), while Ekuni et al. [51] found that the impacts attributed to malocclusion significantly contributed to psychological stress (β = 0.18, *p* < 0.001). Table 6 presents a review of the studies that are dedicated to both anxiety and depression.

## 4. Discussion

In this review, we studied the potential association between mental health implications—in particular, between anxiety and depression and skeletal or dental malocclusion. A total of 11 studies were included in this review, three of which focused on anxiety and dental malocclusion [45,46,47], while one examined the relationship between anxiety and skeletal malocclusion [13]. Similarly, depression in relation to dental malocclusion was analyzed in two studies [48,55]. One study [49] explored both anxiety and depression in relation to skeletal malocclusion. Meanwhile, five studies examined the association between anxiety, depression, and dental malocclusion [49,50,51,52,54]. The majority of the 11 studies (36.36%) investigated both anxiety and depression in relation to dental malocclusion. Anxiety and dental malocclusion were explored in 27.27% of the studies, while depression and dental malocclusion were explored in 18.18%. Skeletal malocclusion received less attention, with only 9.09% of studies focusing on anxiety and another 9.09% examining both anxiety and depression.

Relationship between anxiety and malocclusion:

Metin-Gürsoy et al. [45] assessed 431 adolescents (>12 years; 64.2% female) using the STAI-T and CDAS, revealing 34.1% had severe anxiety, with higher malocclusion severity (ICON scores) strongly linked to elevated anxiety (*p* ≤ 0.0001). Silva et al. [46] studied 199 children (6–14 years) via the IOTN-DHC and HADS, reporting 19.6% had anxiety and 20% had oral habits (e.g., thumb sucking) independent of anxiety. Atik et al. [13] analyzed 120 patients (median age ~14–15) with Class I–III malocclusions, finding Class II/III groups had significantly higher levels of social appearance anxiety (SAAS) and sensitivity to criticism scores, correlating with malocclusion complexity (ICON, *r =* 0.247). Reshitaj et al. [47] compared 127 children (11–14 years; 66% female) with malocclusion or caries, noting lower dental anxiety in malocclusion groups (8.86 vs. 10.80, *p* < 0.001) but higher anxiety in girls overall. Collectively, severe malocclusion (Class II/III) and aesthetic concerns amplify anxiety, particularly in adolescents, while gender and oral habits further modulate psychological impacts.

Relationship between depression and malocclusion:

Two studies [49,50] explored depression and malocclusion, revealing mixed outcomes. The former study (*n* = 350) identified a higher depression prevalence in Class III malocclusions (39.6%) versus Class I (22.4%) patients using the BDI (*p* = 0.003). In contrast, Alsulaiman et al. [55] (*n* = 3806) found no significant link between malocclusion and major depressive episodes but noted dysthymia was associated with upper crowding (DSM-III criteria).

Relationship between anxiety, depression, and malocclusion:

Broader psychological impacts were examined using diverse instruments. Koskela et al. [54] (*n* = 2076) found no significant anxiety/depression differences between patients with severe malocclusion and controls via the HADS and STAI (*p* = 0.378–0.190). Medvedev et al. [49] (*n* = 42) reported higher anxiety in cleft/non-cleft malocclusion groups (34.7% and 29.6%) versus controls (18.7%) using the HADS. Zhang et al. [50] (*n* = 348) in China linked malocclusion to elevated anxiety/depression via Symptom Checklist 90 (SCL-90; *p* < 0.001), with a correlation with neuroticism (EPQ-N). Ekuni et al. [51] (*n* = 641) in Japan demonstrated malocclusion-related impacts on interpersonal sensitivity and depression (Hopkins Symptom Checklist; β = 0.92, *p* < 0.001). These findings emphasize malocclusion’s multifaceted psychological burden, assessed through validated psychometric tools.

Assessing quality and risk of bias:

To assess the methodological quality and the risk of bias, the JBI checklist was used. The JBI methodology provides comprehensive evidence synthesis, integrating diverse study types for a holistic understanding of clinical issues [89]. Its rigorous critical appraisal processes enhance the reliability of findings, and its adaptability to various research questions makes it versatile across healthcare domains. The JBI checklist’s focus on evidence-based practice aims to improve patient outcomes through informed decision making, and its extensive support resources empower researchers to conduct transparent, credible, and clinically relevant reviews, making it valuable for advancing evidence-based healthcare.

Methin-Gürsoy et al. [45] was the only one out of the four studies on anxiety and dental malocclusion to meet almost all quality standards; the other three’s descriptions of the study conditions and their handling of confounding variables were ambiguous. Rafiei et al. [48] study had an emphasis on depression and performed well in terms of outcome reporting and exposure measurement but performed poorly in other quality dimensions. Research addressing anxiety and depression together (*n* = 5) performed well in terms of inclusion criteria, outcome evaluation, and research design but often failed to identify and address confounding variables. The quasi-experimental study by Azuma et al. [52] met most standards, although it lacked a control group and several outcome assessments. The cohort study by Koskela et al. [54] showed mixed results, with strengths in terms of group comparability and exposure assessment, but weaknesses in terms of follow-up completeness and strategies to address this incomplete follow-up. Overall, while most studies demonstrated some methodological strengths, there were consistent gaps across the studies in addressing confounding factors and, in some cases, in clearly defining study parameters or ensuring a comprehensive follow-up. Table 1, Table 2 and Table 3 provide quality appraisal according to the JBI criteria.

## 5. Limitations

This review’s primary drawback is the small number of studies it considered, all of which had a moderate-to-high risk of bias. However, this is justified by the lack of research that examines the direct relationship between malocclusion (i.e., as a predictor variable) and anxiety or depression (i.e., as outcome variables). Therefore, the level of confidence in the results has been impacted by this. Additionally, differences in malocclusion type, anxiety or depression evaluation methods, and assessment times across the included studies were another challenge for this review. Therefore, it was not possible to combine the data into a meta-analysis that would have yielded a reliable estimate of the treatment impact. Furthermore, it was not possible to confirm the impact of age or gender on patients in any of the included studies; therefore, additional research is required to provide strong evidence in this area.

## 6. Conclusions

The modest body of literature provides moderate-quality evidence suggesting a positive correlation between both anxiety and depression and skeleto-dental malocclusion. Notably, investigations specifically addressing the correlation between depression and skeleto-dental malocclusion remain scarce. Some studies show higher rates of psychological distress in individuals with malocclusion, while others find no significant difference. Variations in study design, assessment methods, and population characteristics are likely to contribute to these inconsistent results. Further research using standardized measures and considering factors such as malocclusion severity and cultural contexts is necessary to clarify this relationship. Until more conclusive evidence emerges, clinicians should consider potential psychological impacts on a case-by-case basis when treating malocclusion.

## Figures and Tables

**Table 1:** Evaluation of quality and risk of bias conducted according to JBI Critical Appraisal Checklist for Analytical Cross-Sectional Studies [42] for studies on relationship between anxiety and skeleto-dental malocclusion.

Study	Inclusion Criteria Clearly Defined?	Proper Study Subjects and the Setting Description?	Was the Exposure Measured in a Valid and Reliable Way?	Objective, Standard Criteria Used for Measurement of the Condition?	Confounding Factors Identified?	Strategies to Deal with Confounding Factors Stated?	Outcomes Measured in a Valid and Reliable Way?	Appropriate Statistical Analysis Used?
Metin-Gürsoy et al. [45]	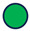	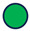	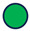	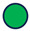	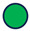	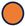	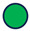	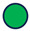
Silva et al. [46]	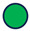	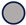	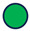	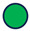	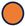	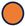	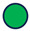	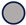
Atik et al. [13]	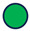	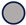	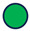	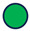	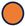	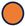	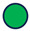	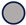
Reshitaj et al. [47]	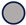	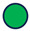	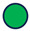	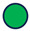	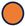	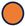	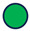	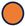

NOTE: 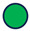 = Yes, 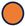 = No, 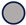 = Unclear. Metin-Gürsoy et al. [45]: 7 “Yes” out of 8 = 87.5%; overall low risk. Silva et al. [46]: 4 “Yes”, 2 “No”, and 2 “Unclear” out of 8 = 50% “Yes”; overall moderate risk. Atik et al. [13]: 4 “Yes”, 2 “No”, and 2 “Unclear” out of 8 = 50% “Yes”; overall moderate risk. Reshitaj et al. [47]: 4 “Yes”, 3 “No”, and 1 “Unclear” out of 8 = 50% “Yes”; overall moderate risk.

**Table 2:** Evaluation of quality and risk of bias conducted according to JBI Critical Appraisal Checklist for Analytical Cross-Sectional Studies [42] for studies on relationship between depression and skeleto-dental malocclusion.

Study	Inclusion Criteria Clearly Defined?	Proper Study Subjects and the Setting Description?	Was the Exposure Measured in a Valid and Reliable Way?	Objective, Standard Criteria Used for Measurement of the Condition?	Confounding Factors Identified?	Strategies to Deal with Confounding Factors Stated?	Outcomes Measured in a Valid and Reliable Way?	Appropriate Statistical Analysis Used?
Rafiei et al. [48]	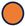	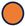	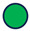	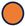	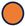	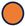	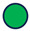	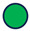
Alsulaiman et al. [55]	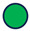	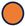	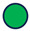	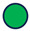	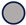	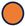	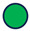	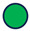

NOTE: 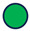 = Yes, 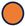 = No, 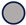 = Unclear. Rafiei et al. [48]: 3 “Yes”, 5 “No”, and 0 “Unclear” out of 8 = 37.5% “Yes”; overall high risk. Alsulaiman et al. [55] 5 Yes”, 2 “No”, and 1 “Unclear” out of 8 = 37.5% “Yes”; overall moderate risk.

**Table 3:** Evaluation of quality and risk of bias conducted according to JBI Critical Appraisal Checklist for Analytical Cross-Sectional Studies [42], Quasi-Experimental Studies [36], and Cohort Studies [36] on relationship between anxiety, depression, and skeleto-dental malocclusion.

Study	Inclusion Criteria Clearly Defined?	Proper Study Subjects and Setting Description?		Was the Exposure Measured in a Valid and Reliable Way?	Objective, Standard Criteria Used for Measurement of the Condition?	Confounding Factors Identified?	Strategies to Deal with Confounding Factors Stated?	Outcomes Measured in a Valid and Reliable Way?		Appropriate Statistical Analysis Used?	
Medvedev et al. [49]	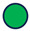	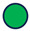		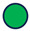	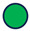	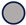	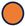	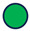		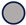	
Zhang et al. [50]	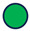	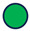		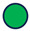	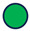	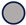	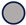	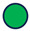		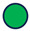	
Ekuni et al. [51]	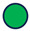	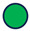		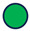	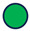	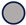	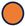	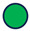		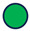	
Evaluation of quality and risk of bias conducted according to JBI Checklist for Quasi-Experimental Studies Studies [36] for studies on relationship between anxiety, depression, and skeleto-dental malocclusion											
Study	Is it clear in the study what is the cause’ and what is the ‘effect’?	Were the participants included in any comparisons similar?	Were the participants included in any comparisons receiving similar treatment/care, other than the exposure or intervention of interest?	Was there a control group?	Were there multiple measurements of the outcome both pre and post the intervention/exposure?	Was follow up complete, and if not, were differences between groups in terms of their follow up adequately described and analyzed?	Were the outcomes of participants included in any comparisons measured in the same way?	Were outcomes measured in a reliable way?	Was appropriate statistical analysis used?		
Azuma et al. [52]	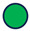	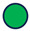	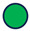	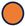	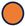	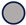	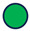	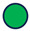	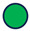		
Evaluation of quality and risk of bias conducted according to JBI Checklist for Cohort Studies [36] for studies on relationship between anxiety, depression, and skeleto-dental malocclusion											
Study	Were groups comparable and from same population?	Was exposure assessment consistent across groups?	Was exposure measurement valid and reliable?	Were confounders identified?	Were methods to address confounding specified?	Were participants outcome-free at baseline?	Was outcome measurement valid and reliable?	Was follow-up duration adequate and reported?	Was follow-up complete? If not, were reasons for loss explored?	Were strategies used to address incomplete follow-up?	Was the statistical analysis appropriate?
Koskela et al. [54]	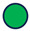	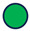	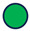	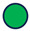	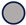	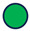	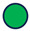	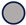	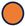	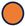	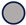

NOTE: 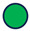 = Yes, 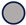 = No, 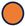 = Unclear. Medvedev et al. [49]: 5 “Yes”, 1 “No”, and 2 “Unclear” out of 8 = 62.5% “Yes”; overall moderate risk. Zhang et al. [50]: 6 “Yes”, 0 “No”, and 2 “Unclear” out of 8 = 75% “Yes”; overall moderate risk. Ekuni et al. [51]: 6 “Yes”, 1 “No”, and 1 “Unclear” out of 8 = 75% “Yes”; overall moderate risk. Azuma et al. [52]: 6 “Yes”, 2 “No”, and 1 “Unclear” out of 9 applicable criteria = 66.67% “Yes”; overall moderate risk. Koskela et al. [54]: 6 “Yes”, 2 “No”, and 3 “Unclear” out of 11 applicable criteria = 54.55% “Yes”; overall moderate risk.

**Table 4:** A review of studies, their findings, and implications on correlation between anxiety and malocclusion.

Study Details	Sample Size (*n*)	Population, Age (Years), and Gender	Psychological Assessment	Assessment Methods	Outcome
Authors: Metin-Gürsoy et al. [45] Year: 2023 Country: Turkey	*n* = 431	Orthodontic; Adolescent; Over 12; 35.8% male 64.2% female	Social anxiety	STAI-T; CDAS	A total of 38.28% had mild anxiety, 34.1% severe anxiety, and 27.62% moderate anxiety; CDAS scores were significantly lower in mild anxiety group compared to moderate and severe groups (*p* ≤ 0.0001); ICON scores significantly higher in severe anxiety group (*p* ≤ 0.0001); Positive correlation between STAI-T and both CDAS and ICON scores
Authors: Silva et al. [46] Year: 2021 Country: Brazil	*n* = 199	Mixed-dentition, Permanent-dentition, non-orthodontic; 6–14;	Anxiety; malocclusion; oral habits	IOTN-DHC [78], HADS [63]; Oral habits questionnaire	Prevalence of anxiety: 19.6%; 20% of schoolchildren displayed oral habits regardless of their anxiety status;
Authors: Atik et al. [13] Year: 2021 Country: Turkey	*n* = 120	malocclusions (Class I, II, and III); (Median ages): Group 1: 14 Group 2: 14.25 Group 3: 15.15;	Anxiety; complexity	ICON [79]; SAAS [80]; STCS	Groups 2 (Class II) and 3 (Class III) had significantly higher SAAS scores compared to Group 1 (Class I); Group 2 (Class II) had significantly higher STCS scores; Positive correlation between ICON and SAAS scores (*r =* 0.247, *p* = *0*.007);
Authors: Reshitaj et al. [47] Year: 2019 Country: Kosovo	*n* = 127	Orthodontic, dental caries, malocclusion; 11–14; 34% male and 66% female	Dental anxiety	CDAS	Dental anxiety was lower in malocclusion group (8.86 ± 2.78) compared to caries group (10.80 ± 2.75) (*p* < 0.001); Girls showed higher anxiety levels in both groups (*p* < 0.001)

**Table 5:** A review of studies and their findings of and implications for correlation between depression and malocclusion.

Study Details	Sample Size (*n*)	Population, Age (Years), and Gender	Psychological Assessment	Assessment Methods	Outcome
Authors: Rafiei et al. [48] Year: 2020 Country: Iran	*n* = 350	Orthodontic, malocclusions (Class I, II, and III); 16–29; 70.6% female 29.4% male	Depression	Angle’s classification; Orthodontist examination; BDI; Chi-square, One-way ANOVA, *t*-test, Tukey’s test, SPSS v23	Overall depression prevalence: 28.3%; Class I: 22.4% depression prevalence; Class II: 32.7% depression prevalence; Class III: 39.6% depression prevalence; Class III patients had significantly higher levels of depression than Class I (*p* = 0.003); No significant association between depression and gender, marital status, or educational status (*p* > 0.05);
Authors: Alsulaiman et al. [55] Year: 2024 Country: KSA	*n* = 3806	Nationally representative sample of young adults from the United States; 18–39 years; 50.68% female 49.32% male	Depression	Structured interviews based on DSM-III criteria conducted by trained interviewers within MECs	Logistic regression models found no significant association between malocclusion traits and major depressive episodes (MDEs). However, the models revealed a positive association between malocclusion traits (specifically, upper crowding and two or more cumulative malocclusion traits) and dysthymia.

**Table 6:** A review of studies and their findings of and implications for correlation between anxiety, depression, and malocclusion.

Study Details	Sample Size (*n*)	Population, Age (Years), and Gender	Psychological Assessment	Assessment Methods	Outcome
Authors: Azuma et al. [52] Year: 2008 Country: Japan	*n* = 31	Orthodontic; 17.3–42.5 (Mean: 25.4);	Anxiety; Depression; Oral health; Facial satisfaction	STAI [59]	STAI-I significantly decreased after surgery (*p* < 0.01); No significant change in trait anxiety (STAI-II);
Authors: Koskela et al. [54] Year: 2021 Country: Finland	*n* = 2076	Adolescents with severe malocclusion and controls; 16 years; Boys 50%, girls 50%	Attention deficit hyperactivity disorder (ADHD); Asperger’s syndrome; Autism; Mood disorders; Broadly defined behavioral abnormalities; Learning problems; Sleep disturbances; Anxiety symptoms; Depressive symptoms	TPI; HADS; STAI; Pearson’s chi-squared test; two-sided *t*-tests	Anxiety disorders were observed in 4.5% (93) of the total research group. Of these, 2.0% (41) were in the study group (severe malocclusion) and 2.5% (52) were in the control group. The difference was not statistically significant (*p* = 0.378); Depression was observed in 3.9% (82) of the total research group. Of these, 1.6% (34) were in the study group and 2.3% (48) were in the control group. Again, this difference was not statistically significant (*p* = 0.190).
Authors: Medvedev et al. [49] Year: 2017 Country: Russia	*n* = 42	Cleft patients and non-cleft patients with skeletal Class II, Class III, and anterior open bite malocclusions; 24 ± 7.2 years (mean); 78.6% female 21.4% male		IOTN-DHC [80]; HADS; STAI	Cleft patients (1st group): Anxiety symptoms: 34.7% Depression symptoms: 17.2% High rates of reactive anxiety: 35.8% Non-cleft patients with skeletal malocclusions (2nd group): Anxiety symptoms: 29.6% Depression symptoms: 13.1% High rates of reactive anxiety: 34.2% Control group (3rd group): Anxiety symptoms: 18.7% Depression symptoms: 8.3% High rates of reactive anxiety: 17.7%
Authors: Zhang et al. [50] Year: 2012 Country: China	*n* = 348	Angle’s Class I, II, and III malocclusion; 18–39 years (mean age: 25.5 ± 3.5 years); 173 males (49.7%); 175 females (50.3%)	Obsessive-compulsiveness, Interpersonal sensitivity, Depression, Anxiety, and Paranoid ideation.	SCL-90; EPQ); IOTN	Patients with Angle’s Class I, II, and III malocclusion exhibited significantly higher scores on anxiety and depression subscales of the SCL-90 compared to the normal occlusion group (*p* < 0.001); No significant differences were found among the Class I, II, and III malocclusion groups in terms of anxiety and depression scores; A significant positive correlation was observed between neuroticism (EPQ-N) scores and all SCL-90 subscales, including anxiety and depression (*p* < 0.01).
Authors: Ekuni et al. [51] Year: 2011 Country: Japan	*n* = 641	Orthodontic, no systemic diseases or drug consumption in the previous 2 months), non-smokers, non-pregnant; 18–19 years; 51.34% males; 48.66% females	Somatization; Obsessive–compulsiveness; Interpersonal sensitivity; Anxiety; Depression;	IOTN; HSCL	Subjects with impacts on daily performance attributed to malocclusion had significantly higher Hopkins Symptoms Checklist (HSCL) scores, indicating higher levels of psychological stress; Using structural equation modeling (SEM), the study found that impacts attributed to malocclusion contributed to psychological stress (β = 0.18, *p* < 0.001); The impacts were particularly conducive to interpersonal sensitivity (β = 0.92, *p* < 0.001) and depression (β = 0.92, *p* < 0.001).

## Data Availability

No datasets were generated or analyzed during the current study.
